# Iranian nurses' knowledge and attitude toward complementary and alternative medicines: Is there any relation with quality of nursing care?

**DOI:** 10.3389/fpubh.2022.942354

**Published:** 2022-08-15

**Authors:** Mahlagha Dehghan, Zakieh Namjoo, Mahlasadat Jafari, Ghazaleh Kordestani, Nazanin Tabebordbar, Fatemeh Payam, Mohammad Ali Zakeri, Sima Mokhtarabadi

**Affiliations:** ^1^Nursing Research Center, Kerman University of Medical Sciences, Kerman, Iran; ^2^Student Research Committee, School of Nursing and Midwifery, Kerman University of Medical Sciences, Kerman, Iran; ^3^Social Determinants of Health Research Center, Rafsanjan University of Medical Sciences, Rafsanjan, Iran; ^4^Shafa Hospital, Kerman University of Medical Sciences, Kerman, Iran

**Keywords:** knowledge, attitude, complementary and alternative medicine, quality of nursing care, nurse

## Abstract

**Background:**

Nurses play an important role in advising and guiding patients on effective treatments, and in this situation, it is better to be aware of complementary and alternative medicine (CAM) as well as the effects and side effects of different procedures. In addition, the quality of nursing care is directly related to the nurse's knowledge of the different treatments and preventions of different diseases and conditions. The present study aimed to investigate Iranian nurses' knowledge and attitude toward complementary and alternative medicines (CAMs) as well as their correlation with the quality of patient care.

**Materials and methods:**

This cross-sectional study included 267 clinical nurses from three hospitals in southern Iran. The participants were recruited using convenience sampling methods in 2020–2021. A demographic questionnaire, knowledge and attitude toward CAM questionnaires, and the Quality Patient Care Scale were used to collect data.

**Results:**

The mean score of CAM knowledge was 26.51, which was less than the questionnaire midpoint of 39. The mean score of attitudes toward CAM was 63.84, which was more than the questionnaire midpoint of 57. The mean score of the quality of patient care was 197.80, which was more than the questionnaire midpoint of 130. There was no significant correlation between knowledge about CAM, quality of patient care, and its dimensions. In addition, there was no significant correlation between attitudes toward CAM, quality of patient care, and its dimensions (*P* > 0.05).

**Conclusion:**

The results of the present study showed that nurses' attitudes toward the use of CAM are positive and can be a sign of their readiness to use and further integrate clinically approved CAM in patient nursing care. Due to the role of nurses in patient care and treatment, there is a need to increase nurses' knowledge of CAM, and its training should be included in the nursing curriculum. More studies are needed to identify nurses' knowledge and attitudes toward CAM and its impact on the quality of nursing care.

## Introduction

Nurses make up the largest part of the professional staff, in other words, accounting for roughly 70% of the healthcare team responsible for providing services in the health care system (1). Given that nurses play a critical role in clinical care, service responsiveness, and have various responsibilities involving direct care and health education of patients, nurses are considered a major health professional group in healthcare delivery (2). Any deficiency in this group will affect the quality and quantity of health services and, ultimately, the health of the community (3). Nurses, as caregivers, play a major role in improving and enhancing the quality of health care by assessing, planning, and evaluating patient care needs (4). The quality of nurses' care is affected by several factors that can affect the safety and improvement of patients' clinical condition (5). One of these factors is the knowledge and attitude of nurses toward CAM that should be considered (6). The World Health Organization defines complementary medicine as “knowledge, skills, and practices based on indigenous theories, beliefs, and experiences from other cultures, which are used to maintain health, prevent disease, and promote recovery or physical and mental therapy (7, 8). Today, the world is experiencing a surge in public demand and the use of various CAM methods, as well as the number of referrals to specialists and the cost of CAM treatments for the treatment of diseases and health promotion (9).

The people of Iran pay special attention to the use of CAM, especially due to the prevalence of diseases and crises, the use of CAM has increased (10). A high percentage of the use of CAM has been reported in Iranian patients, such as cancer (87.3%) (11), diabetes (75.3%) (12) hemodialysis (75.2%) (13) and thalassemia patients (68.5%) (14) which can be due to the ease of use and popularity of CAM in Iranian society. In addition, these patients more frequently are contacted with nurses in the hospital and get advice from them. Nurses play an important role in advising and helping patients toward effective treatments. Therefore, they must have sufficient knowledge about CAM, as well as the effects and side effects of different methods of CAM (15, 16).

Nurses often deal with a large number of patients who use CAM therapies (17) and can be a valuable source of information about CAM treatments for patients (18). On the other hand, supporting nurses to use CAM is not an attempt to challenge conventional therapies but an attempt to improve the quality of patient care (19). There are many barriers to supporting CAM because nurses have insufficient training in this area (17). Chang et al. (6) in Taiwan point to barriers to supporting CAM such as organizational culture, clinical framework, and time and knowledge constraints in the nursing profession. As the results of studies on nurses' knowledge and attitudes toward the use of complementary medicine are very diverse, nurses' knowledge about different methods of CAM, particularly acupressure, relaxation and touch therapy, massage therapy, and reflexology, has been reported very little (15–17). However, Källman et al. (20) and Strand and Lindgren's (21) in Sweden found that the knowledge of nurses was favorable. In addition to variable knowledge about CAM treatments, nurses often have different attitudes toward the patient's use of such procedures (22). Despite the fact that these techniques are not used in clinical care, there is a positive attitude and an interest in learning them (17). Professional ideology, knowledge, and the field in which nurses work all affect their attitudes toward CAM (23). In the study, Tracy et al. (24) showed that training programs that provide information about the use of complementary and alternative therapies and evidence-based therapies are likely to increase the appropriate use of therapies to achieve optimal treatment outcomes. The study by Tagharrobi et al. (25) showed that the context of Iranian clinical settings is suitable for the use of complementary therapies in nursing practice. These potential opportunities for the use of complementary therapies in clinical settings can be associated with beneficial effects for patients.

Cooke et al. (26) in Australia showed that 88% of nurses who participated in the study used CAM and became more familiar with these techniques. The main motivations for nurses' usage of CAM were to reduce stress and anxiety and improve health (27). The use of CAM therapies in patients requires careful evaluation by nurses and other professionals in order to be safe, effective, and functional and to protect vulnerable patients (8). Therefore, the knowledge and attitude of nurses toward using CAM therapies is very important. Therefore, this study was conducted to investigate the knowledge and attitudes of nurses toward CAM as well as its relationship with the quality of nursing care.

## Materials and methods

### Study design and setting

This cross-sectional study was conducted in three hospitals in Kerman, Iran. Kerman is the largest city in the southeast of Iran, with a population of more than 722,000 (28).

### Sampling and sample size

Sampling was done using the convenience sampling method. Inclusion criteria were informed consent, having a bachelor's degree in nursing or higher, nurses in any specialty, and nurses with more than 1 year of service experience. The exclusion criterion was questionnaires that had been filled in incompletely. The sample size was estimated to be *n* = 267 using the Cochran formula (α = 0.05, *d* = 0.06, *Z* = 1.96) (29). Considering dropout probability, 300 questionnaires were distributed, of which 33 questionnaires were excluded from the study due to a high rate of missing values. Therefore, the effective response rate was 89%.

### Instrument

Data were collected using four questionnaires, including socio-demographic form, knowledge about CAM questionnaire, the attitude toward CAM questionnaire, and the quality of patient care scale.

#### Socio-demographic form

According to the literature in the fields of quality of nursing care and nurses' knowledge and attitude toward CAM and similar studies, socio-demographic variables were prepared. This form includes gender, age, marital status, educational level, job history, hospital, ward, history of training about CAM, and using CAM in care.

#### Knowledge about the CAM questionnaire

This questionnaire has been designed by Zeighami and Soltaninejad (17) for Iranian nurses (Persian language). The questionnaire consists of 13 items about the nurses' knowledge and familiarity with different CAM methods, including acupuncture, acupressure, touch therapy, energy therapy, nutrition therapy, herbal medicine, music therapy, hydrotherapy, massage, relaxation, relaxation, yoga, and hypnosis. These questions are graded on a five-point Likert scale (lack of knowledge = 0 to very high knowledge = 4). The final score of knowledge about CAMs is between 0 and 52, with a higher score indicating higher knowledge about CAMs (17).

In Iran, in Zeighami and Soltaninejad's study, the content validity of the items of knowledge was 0.98 and the reliability of this questionnaire was reported from the appropriate internal consistency reliability method and Cronbach's alpha coefficient for the items of knowledge, was 0.91 (17). In the present study, the Cronbach's alpha coefficient was 0.92.

#### Attitude toward CAM questionnaire

This questionnaire has been designed by Zeighami and Soltaninejad (17) for Iranian nurses (Persian language). The questionnaire consists of 19 items on a five-point Likert scale (from strongly disagree = 0 to strongly agree = 4). The final score for attitude toward CAM is between 0 and 76, with a higher score indicating a more positive attitude toward CAM. The items of this questionnaire were about the attitude of nurses toward the importance and effect of CAM in managing and caring for patients. The content validity index of attitudes toward the CAM questionnaire in the study of Zeighami and Soltaninejad was 0.97. In addition, the internal consistency method was used to assess the reliability of this questionnaire, and Cronbach's alpha coefficient was reported to be 0.98 (17). In the present study, the Cronbach's alpha coefficient was 0.78.

#### Quality patient care scale

The Quality Patient Care Scale (Qualpacs) is another American tool published in 1974, the result of the collaboration of two professors, Wandelt and Ager, along with faculty members at Wayne State University's College of Nursing (30). This questionnaire has 65 questions divided into three dimensions: psychosocial (28 questions), communication (13 questions) and physical (24 questions). This tool is on the five-point Likert scale (no case: 0, rarely: 1, sometimes: 2, most of the time: 3, always: 4). The scores on this questionnaire range from 0 to 260. Scores of 0–136 are interpreted as unpleasant, scores of 137–204 are interpreted as partly unpleasant, and scores of 205–260 are interpreted as pleasant.This questionnaire is one of the most reliable tools for measuring the quality of patient care (31). Haghighi Khoshkho examined the validity of this tool in Iranian nurses (32). So far, its validity has been confirmed and it has been used in various studies (33, 34). The reliability of the quality of patient care scale has also been confirmed in several studies (32, 33, 35). In the present study, the Cronbach's alpha coefficient was 0.96.

### Data collection and ethical considerations

In the present study, the target population consisted of nurses in three educational hospitals affiliated with Kerman University of Medical Sciences. After obtaining the code of ethics from the ethics committee of Kerman University of Medical Sciences (IR.KMU.REC.1399.437), the researcher referred to the research setting and the license was given to the managers of hospitals (Shafa, Bahonar, and Afzalipour). The purpose of the study and how to fill out the questionnaires were explained to the participants, and the eligible samples who were willing to participate in the study were asked to carefully complete the questionnaires. The researchers went to the relevant centers to collect data during all three shifts of the morning, evening, and night. When the ward's workload was light, questionnaires were distributed to nurses. Questionnaires were collected at the same time or, if necessary, at the end of the shift. Samples were assured that all information would remain confidential. The sampling lasted from December 2020 to May 2021.

### Data analysis

Statistical Package of Social Sciences for Windows Version 22 (SPSS-22) was used to analyze the data. The data was described using descriptive statistics (frequency, percentage, mean, and standard deviation). Since the study's main variables had normal distribution according to the Kolmogorov-Smirnov test, the Pearson correlation coefficient was used to determine the correlation between the knowledge and attitudes about CAM and the quality of patient care. Based on the quantitative variables of the study, an independent *t*-test and an analysis of variance test were used to determine knowledge and attitudes toward CAM and the quality of patient care. The significance level of 0.05 was used.

## Results

The mean age of the participants was 33.90 ± 8.66 (Min = 21 and Max = 55). The majority of the participants were female, married, and had a bachelor's degree. The work experience was between one and 32 years (Mean = 10.15, SD = 8.13). The highest frequency of work experience was 5 years (Table 1).

**Table 1:** Participants characteristics and the association between participants' characteristics and knowledge of CAM, attitudes toward CAM, and quality of nursing care (*n* = 267).

**Variables**	**Frequency (Valid percent)**	**Knowledge of CAM**		**Attitudes toward CAM**		**Quality of patient care**	
		**Mean (SD)**	**Statistical test (*P* value)**	**Mean (SD)**	**Statistical test (*P* value)**	**Mean (SD)**	**Statistical test (*P* value)**
**Gender**							
Male	21 (7.9)	29.38 (10.53)	*t* = −1.31 (0.19)	63.14 (6.54)	*t* = 0.42 (0.67)	192.9 (37.03)	*t* = 0.79 (0.43)
Female	246 (92.1)	26.26 (10.50)		63.90 (7.92)		198.22 (28.79)	
**Age (yr.)**							
≤ 30	120 (44.9)	27.47 (10.45)	*t* = 1.35 (0.18)	63.44 (7.82)	*t* = −0.75 (0.45)	194.94 (30.62)	*t* = −1.43 (0.15)
>30	147 (55.1)	25.72 (10.56)		64.16 (7.81)		200.13 (28.38)	
**Marital status**							
Unmarried	85 (31.8)	28.2 (10.15)	*t* = 1.80 (0.07)	63.98 (7.88)	*t* = 0.20 (0.84)	195.13 (28.84)	*t* = −1.01 (0.31)
Married	182 (68.2)	25.71 (10.63)		63.77 (7.80)		199.04 (29.75)	
**Educational level**							
Bachelor's	234 (87.6)	26.29 (10.55)	*t* = −0.91 (0.37)	63.51 (7.53)	*t* = −1.82 (0.07)	199.04 (29.38)	*t* = 1.85 (0.07)
Master's	33 (12.4)	28.06 (10.40)		66.15 (9.37)		188.97 (29.04)	
**Work experience (yr.)**							
>5	106 (39.7)	27.73 (10.09)	*F* = 1.34 (0.26)	62.98 (7.11)	*F* = 1.16 (0.32)	197.75 (30.59)	***F*** **=** **2.82 (0.04)**
5–10	41 (15.4)	27.17 (12.32)		65.46 (9.25)		186.71 (28.92)	
11–15	48 (18.0)	26.08 (9.76)		63.52 (7.76)		199.42 (30.65)	
>15	72 (27.0)	24.61 (10.47)		64.39 (7.91)		203.10 (25.99)	
**Hospital**							
A	69 (25.8)	28.48 (10.09)	*F* = 2.61 (0.08)	64.93 (8.29)	*F* = 1.52 (0.22)	197.77 (31.68)	*F* = 1.40 (0.25)
B	100 (37.5)	24.8 (10.43)		62.85 (7.39)		194.34 (28.2)	
C	98 (36.7)	26.86 (10.75)		64.08 (7.84)		201.35 (28.99)	
**Ward**							
Critical/intensive	79 (29.6)	26.51 (11.05)	*F* = 1.70 (0.15)	66.04 (8.62)	***F*** **=** **2.81 (0.03)**	197.05 (27.56)	*F* = 0.62 (0.65)
Medical	77 (28.8)	28.56 (10.14)		62.28 (6.43)		195.86 (30.19)	
Surgical	62 (23.2)	24.29 (10.61)		62.87 (7.37)		201.34 (28.64)	
Emergency	34 (12.8)	27.06 (10.01)		63.35 (8.63)		200.65 (33.26)	
Others	15 (5.6)	23.87 (9.30)		65.33 (7.83)		190.60 (31.35)	
**History of training about CAM**							
Yes	23 (8.6)	32.61 (9.96)	***t*** **=** **2.95 (0.003)**	69.22 (8.84)	***t*** **=** **3.53 (<0.001)**	196.30 (27.99)	*t* = −0.25 (0.80)
No	244 (91.4)	25.93 (10.41)		63.33 (7.53)		197.94 (29.65)	
**Using CAM in caring**							
Yes	28 (10.5)	29.11 (9.36)	*t* = 1.38 (0.17)	68.89 (8.30)	***t*** **=** **3.70 (<0.001)**	194.25 (29.87)	*t* = −0.67 (0.50)
No	239 (89.5)	26.2 (10.63)		63.25 (7.55)		198.21 (29.46)	

*CAM, Complementary and Alternative Medicine; SD, Standard Deviation. Bold values presented have a significant level*.

The mean score of CAM knowledge was 26.51, which was less than the questionnaire midpoint of 39. The mean score of attitudes toward CAM was 63.84, which was more than the questionnaire midpoint of 57. The mean score of the quality of patient care was 197.80, which was more than the questionnaire midpoint of 130 (Table 2). In addition, 79% (*n* = 211) of the participants rated the quality of patient care as high, while 21% (*n* = 56) rated it as moderate.

**Table 2:** The distribution of knowledge of CAM score, attitudes toward CAM score, and quality of nursing care and its dimensions' scores (*n* = 267).

**Variables**	**Minimum**	**Maximum**	**Mean**	**SD**
Knowledge of CAM	13	55	26.51	10.52
Attitudes toward CAM	51	93	63.84	7.81
Quality of patient care	123	260	197.80	29.47
Psycho-social	42	112	82.44	13.55
Communication	13	52	38.97	7.36
Physical	32	96	76.39	14.01

There was no significant correlation between knowledge of CAM, quality of patient care, and its dimensions. In addition, there was no significant correlation between attitudes toward CAM and the quality of patient care and its dimensions (Table 3).

**Table 3:** The correlation between knowledge of CAM, attitudes toward CAM, and quality of patient care and its dimensions (*n* = 267).

**Variables**	**Knowledge of**		**Attitudes toward**	
	**CAM**		**CAM**	
	***r***	***P* value**	***r***	***P* value**
Quality of patient care	−0.05	0.43	0.05	0.38
Psycho-social	−0.06	0.32	−0.02	0.80
Communication	−0.06	0.35	0.08	0.21
Physical	−0.01	0.09	0.09	0.16

*r = Pearson Correlation Coefficient*.

Among the study variables, only participants who had a history of CAM training had greater knowledge of CAM than the others (*P* = 0.003). In addition, participants who served in critical/intensive units had a more positive attitude toward CAM in comparison with other participants. Participants with a history of CAM training and use of CAM in care had greater knowledge of CAM than others (*P* < 0.05). There was no significant association between study variables and quality of patient care (*P* > 0.05) (Table 1).

## Discussion

### Knowledge about CAM

The results of the present study showed that the mean score of nurses' knowledge of CAM was 12.49 less than the questionnaire midpoint of 39. Consistent with the results of the present study, in a study by Shorofi et al. (36), 29.7% of the participants reported knowledge about CAM treatments. Shorofi et al. (37) found that more than 60% of nurses had little or no knowledge of CAM. Nurses also had the least knowledge of acupressure, sedation, and touch therapy (17). Also, Görücü and Sayilan (38) found that nurses in Turkey lacked information about CAM treatments due to insufficient training in this field. Peprah et al. (39) also found trained midwives have very limited knowledge about CAM in rural Ghana. However, the results of the following studies are inconsistent with the results of the present study and a higher percentage of awareness is reported. In a systematic review study involving 21 cross-sectional articles, Balouchi et al. (27) showed that 62.2 percent of nurses were knowledgeable with CAM treatments. Holroyd et al. (40) reported that 93.6% of nurses in Hong Kong had knowledge about CAM treatments. The reason for the difference between the present study and the above studies can be attributed to the differences in data collection tools, organizational culture, and clinical context of the study environment. Since the results of the above studies are very diverse, it can be concluded that cultural, religious, educational, and clinical environmental policies in different parts of the world affect the knowledge of the use of complementary medicine methods.

### Attitude toward CAM

The results of the present study showed that the mean score of CAM attitude was 6.84 higher than the questionnaire midpoint of 57. Numerous studies are consistent with the results of the present study (17, 27, 41).

According to a study conducted by James et al. in Sierra Leone, final year students in the three disciplines of medicine, pharmacy, and nursing have a positive attitude toward CAM therapy, with medical students having a more positive attitude than pharmacy and nursing students. More than three-quarters (76.6%) of students in all three groups expressed an interest in CAM therapies, preferring to use them (CAM therapy) as an option (41). Zeighami et al. also showed that while most of the nurses under study did not use CAM in clinical care, they had a positive attitude and an interest in learning CAM. 71.1% of nurses believed that CAM was effective in treating the disease and that the most effective ways to improve patients' health and treat the disease included nutrition therapy, music therapy, and herbal medicine (17). Peprah et al. (39) also found trained midwives have positive perceptions about CAM, use CAM therapies frequently and recommend them to women in Ghana. Balouchi et al. (27) conducted a systematic review of 21 studies in Iran and showed that nurses had positive attitudes toward CAM and nearly two thirds of them used CAM therapies for patients. The similarity of the internal and operational environment of the nursing profession is the common feature between the above studies and the present study, so that Balouchi et al. (27) showed that nurses used CAM to reduce stress and anxiety and improve health. However, inconsistent with the results of the present study, Strand and Lindgren's (21) study in Sweden reported that nurses' attitudes toward CAM were weak. The reasons for the difference in the results are due to a lack of experience, a lack of training to get acquainted with CAM treatment methods, a lack of organizational support, time, resources, equipment, and medical support (6, 41). In confirmation of this, the study by Van Rensburg et al. (42) in South Africa showed that while nursing students rarely used complementary medicines personally, they had a positive attitude toward them and believed that patients should be allowed to learn about complementary medicine practices.

### Knowledge and attitude toward CAM and relationship with quality of care

The present study found no significant relationship between knowledge and attitude toward using CAM and the quality of patient care and its dimensions. Consistent with the results of the present study, Beeckman et al. (43) in Belgium did not report a significant relationship between nurses' knowledge and attitudes and the quality of nursing care. Leach et al. (44) in Australia reported that the most common barriers to using complementary medicine in patient care were a lack of knowledge and skills in using complementary medicine, insufficient support in this area, and a lack of time. Therefore, the diversity in the results can be attributed to the social and cultural context of each community; the general acceptance of complementary medicine treatments is also effective in the quality of patient care. Chianca et al. (45) in Brazil showed that the low quality of nursing care in pressure ulcers was due to insufficient knowledge of nurses about CAM treatments (a type of massage therapy). A study by Krug et al. (46) in Germany showed that a high percentage of patients with mental illness and chronic health problems were consulted by natural health practitioners (HPs), so-called “Heilpraktiker,” which indicates the need to pay attention to CAM treatment in primary care and to regulate natural health physicians. In Iran, patients have the most contact with nurses. In order to use CAM properly and increase patient quality of care, it is necessary to pay attention to the knowledge and attitude of nurses from whom patients and caregivers seek counseling. However, some CAM complications such as arrhythmias (47) and vascular complications (48) need to be followed up and reported by nurses. Therefore, appropriate measures must be taken for patient safety and proper care. Although the results of the present study showed no significant relationship between nurses' knowledge and attitudes and patient care, due to the lack of studies in this regard, it is necessary to pay attention to this issue in future studies and in other communities to better understand the factors affecting quality patient care.

### Demographic variables and knowledge and attitudes toward CAM

According to the results of the present study, most of the study participants are married women, with a bachelor's degree and work experience of fewer than 5 years. No significant relationship was found between demographic variables and knowledge and attitudes toward CAM and the quality of nursing care. The results of the present study are in line with those of Hiizil et al. (49) in Turkey, who studied physicians, nurses, patients, and patients' companions, as well as Gungorms et al. (50) in Turkey, who studied patients. Despite the different study populations, the coherence of these studies shows that there is no need to invest in a specific target group to teach the use of CAM, and nurses are responsive to education in any demographic situation. Studies conducted by Hayward et al. (51) on oncology healthcare providers in Canada and Siedlecki (52) on US nurses used training as a factor influencing CAM knowledge. Given that research similar to the present study has been conducted on different medical professions in different societies of the world (53), this issue is still of particular importance (49). It is highly informative to discuss the results of this study with studies of different populations in terms of beliefs and cultures. Researchers from different communities should pay attention and focus on such studies. The problems, limitations, and educational needs of nurses need to be identified more precisely. Principled planning should be done for a better quality of life in the future.

### Implications for practice and future research

The use of CAM has always been common among Iranian patients and other Asian patients in other countries. In the meantime, nurses have an important role in counseling and helping patients in order to effectively treat and continuously respond to the care provided to patients. Nurses often deal with a large number of patients who use CAM therapies and can be a valuable source of information about CAM therapies for patients. Therefore, they should have enough information about CAM as well as the effects and side effects of different CAM methods. However, the professional ideology, knowledge, and field in which nurses work all influence their attitudes toward CAM. Nurses' knowledge and attitude toward using complementary medicine to guide and advise patients in the correct use of CAM has a very important role that may improve the quality of patient care. Future studies need to focus on various factors affecting the knowledge and attitude of nurses and their effects on the quality of patient care.

### Limitations

There are certain limitations to the current study. The current study is a cross-sectional study design with the issue of bi-directionality and a lack of control for confounding factors for the significant relationships. Consequently, the study results should be interpreted in light of these constraints and future studies are needed to address these shortcomings. Because the accuracy of the information depends on the individual's memory and concentration, and its accurate and accurate reporting, the results of the study may be subject to the recall bias. Fatigue may also affect the accuracy of the information when answering questions. The study data was collected from an Iranian city that may not be representative of other cities and provinces. Therefore, generalization of the findings of this study to different geographical locations and different demographic characteristics should be done with caution. Due to the fact that the present study was performed at the time of the outbreak of coronavirus and nurses had a high workload, a small sample size (*d* = 0.06) was used. Therefore, it is necessary to consider larger groups in future studies.

## Conclusions

The results of this study showed poor knowledge of nurses in using some CAM treatments, and it seems that training these people can increase their knowledge of CAM. However, the attitude of nurses toward the use of CAM treatments is positive, which is one of the cornerstones of success in implementing a CAM program in the clinic. Nurses' knowledge and attitudes toward CAM have no effect on the quality of nursing care and its dimensions. On the other hand, in this study, the use of CAM among the participants was not evaluated; therefore, a more comprehensive study on CAM is felt among the nurses from different wards and hospitals. More studies are needed to identify nurses' knowledge and attitudes toward CAM and its impact on the quality of nursing care.

## Data availability statement

The raw data supporting the conclusions of this article will be made available by the authors, without undue reservation.

## Ethics statement

The studies involving human participants were reviewed and approved by Kerman University of Medical Sciences. The patients/participants provided their written informed consent to participate in this study.

## Author contributions

MD, ZN, MJ, GK, NT, FP, and SM develop the study idea and protocol. MD supervised the study sampling and analyzed the data. ZN, MJ, GK, NT, and FP did the sampling. MZ, ZN, MJ, GK, NT, FP, and SM wrote the first draft of the manuscript. All authors read and confirmed the final version of the manuscript.

## Conflict of interest

The authors declare that the research was conducted in the absence of any commercial or financial relationships that could be construed as a potential conflict of interest.

## Publisher's note

All claims expressed in this article are solely those of the authors and do not necessarily represent those of their affiliated organizations, or those of the publisher, the editors and the reviewers. Any product that may be evaluated in this article, or claim that may be made by its manufacturer, is not guaranteed or endorsed by the publisher.

## Abbreviations

ARTs: Assisted Reproductive Technologies
CAM: Complementary and Alternative Medicine
CAMs: Complementary and Alternative Medicines
HPs: Health Practitioners.
